# Barriers to Use of Remote Monitoring Technologies Used to Support Patients With COVID-19: Rapid Review

**DOI:** 10.2196/24743

**Published:** 2021-04-20

**Authors:** Elizabeth Houlding, Kedar K V Mate, Kim Engler, David Ortiz-Paredes, Marie-Pascale Pomey, Joseph Cox, Tarek Hijal, Bertrand Lebouché

**Affiliations:** 1 Chronic Viral Illness Service Royal Victoria Hospital McGill University Health Centre Montréal, QC Canada; 2 Department of Physical Therapy Faculty of Medicine University of Toronto Toronto, ON Canada; 3 Centre for Outcomes Research and Evaluation Research Institute of the McGill University Health Centre Montréal, QC Canada; 4 Centre de recherche du Centre Hospitalier de l’Université de Montréal Montréal, QC Canada; 5 Département de gestion, évaluation et politique de santé École de santé publique de l'Université de Montréal Montréal, QC Canada; 6 Department of Epidemiology and Biostatistics Faculty of Medicine McGill University Montréal, QC Canada; 7 Division of Radiation Oncology McGill University Health Centre Montréal, QC Canada; 8 Department of Family Medicine McGill University Montréal, QC Canada

**Keywords:** remote monitoring, technology, COVID-19, telehealth, asynchronous technology, synchronous technology, mHealth, monitoring, review, barrier, benefit, equity

## Abstract

**Background:**

The COVID-19 pandemic has acted as a catalyst for the development and adoption of a broad range of remote monitoring technologies (RMTs) in health care delivery. It is important to demonstrate how these technologies were implemented during the early stages of this pandemic to identify their application and barriers to adoption, particularly among vulnerable populations.

**Objective:**

The purpose of this knowledge synthesis was to present the range of RMTs used in delivering care to patients with COVID-19 and to identify perceived benefits of and barriers to their use. The review placed a special emphasis on health equity considerations.

**Methods:**

A rapid review of published research was conducted using Embase, MEDLINE, and QxMD for records published from the inception of COVID-19 (December 2019) to July 6, 2020. Synthesis involved content analysis of reported benefits of and barriers to the use of RMTs when delivering health care to patients with COVID-19, in addition to health equity considerations.

**Results:**

Of 491 records identified, 48 publications that described 35 distinct RMTs were included in this review. RMTs included use of existing technologies (eg, videoconferencing) and development of new ones that have COVID-19–specific applications. Content analysis of perceived benefits generated 34 distinct codes describing advantages of RMTs, mapped to 10 themes overall. Further, 52 distinct codes describing barriers to use of RMTs were mapped to 18 themes. Prominent themes associated with perceived benefits included a lower burden of care (eg, for hospitals, health care practitioners; 28 records), reduced infection risk (n=33), and support for vulnerable populations (n=14). Prominent themes reflecting barriers to use of RMTs included equity-related barriers (eg, affordability of technology for users, poor internet connectivity, poor health literacy; n=16), the need for quality “best practice” guidelines for use of RMTs in clinical care (n=12), and the need for additional resources to develop and support new technologies (n=11). Overall, 23 of 48 records commented on equity characteristics that stratify health opportunities and outcomes, including general characteristics that vary over time (eg, age, comorbidities; n=17), place of residence (n=11), and socioeconomic status (n=7).

**Conclusions:**

Results of this rapid review highlight the breadth of RMTs being used to monitor and inform treatment of COVID-19, the potential benefits of using these technologies, and existing barriers to their use. Results can be used to prioritize further efforts in the implementation of RMTs (eg, developing “best practice” guidelines for use of RMTs and generating strategies to improve equitable access for marginalized populations).

## Introduction

Delivering health care in the context of the COVID-19 pandemic is uniquely challenging [1]. The virus is highly contagious, potentially fatal, and symptoms that present early in the course of the illness can be mild and nonspecific [2]. Virulence of SARS-CoV-2 in health settings places health care providers and patients without COVID-19 at increased risk of contracting the illness [2]. The need to provide health care services while limiting in-person interaction places an enormous burden on already overwhelmed health systems.

Some models and technologies for delivering health care remotely existed prior to the COVID-19 pandemic. For instance, video consultation is commonly used to deliver health care services to rural and remote communities [3]. These technologies were used for a diversity of needs, including those who might otherwise have difficulty accessing expertise and health care (eg, due to remote location) [4,5]. Further, more recent public health crises of infectious diseases, including the 2002 severe acute respiratory syndrome epidemic, the 2009 H1N1 pandemic, and the 2015 Ebola epidemic, highlighted the pivotal role remote technologies can play in delivering health care [6-8]. Within a short span of a few weeks, these technologies were at the forefront and were a critical method of offering care services.

To adequately address the challenges of the COVID-19 pandemic, unprecedented deployment of remote monitoring technologies (RMTs) is underway [9,10]. Due to a lack of consensus on the definition of RMTs, we took a broad scope and included any technology that facilitates communication between the health care team and patients [11]. RMTs can be categorized as “synchronous,” where communication occurs in “real time,” such as in videoconferencing, and “asynchronous,” where there is a potential expected time delay in communication, such as email or SMS text message [11,12]. Both these forms of RMT could be delivered through a broad range of devices (eg, app on phone, wearable technology). RMTs at times reflect technology that is commonplace (eg, phone call, app on smartphone) or specialist in purpose (eg, oxygen saturation monitor, specific cell phone app). Further, RMTs have been used for a variety of purposes in health care. This has included monitoring symptoms, illness severity, and adherence to inform and deliver treatment [7].

In the context of the present pandemic, RMTs can be used when delivering health care to those with (eg, monitoring severity of illness in those with the diagnosis) or without diagnoses of COVID-19 (eg, delivering primary health care via telephone to reduce likelihood of COVID-19 transmission). Understanding the use of RMTs for the treatment of COVID-19 is particularly pertinent in the context of an emergent pandemic and will be the focus of the present research.

In an early review of health technologies used to address COVID-19, Ming et al [9] described 110 COVID-19 asynchronous technologies including 17 that monitored patients remotely. The authors reported on the benefits of mobile phone apps, including allowing patients to self-monitor their symptoms and informing triage decisions regarding the need to access tertiary care. This use of RMTs could presumably reduce burden on hospital emergency departments. This study highlighted concerns regarding patient confidentiality and data security when using such mobile apps. Another narrative review described 17 different wearable technologies designed to remotely monitor COVID-19 signs and symptoms [10]. These devices included take-home electrocardiograms, blood pressure monitors, pulse oximeters, thermometers, and stethoscopes. These technologies allowed relevant diagnostic information to be collected. Further, use of these technologies helped optimize patient comfort and convenience while reducing the risk of COVID-19 transmission in the hospital setting and the need for hospital resources to be directed to those with milder symptoms. This review identified challenges in implementation, with a focus on technology-specific limitations—for instance, thermometer inaccuracies due to changes in ambient temperature [10].

As the pandemic develops, there will be waves of new technologies that are developed continuously to meet the emerging challenges. Some of these technologies are here to stay and could be available for future infectious diseases or be part of the routine care delivery model. Specific benefits or disadvantages of RMTs may fluctuate in relevance over time. This paper provides a snapshot that can be used as a baseline to track progress in this field over the course of the pandemic. This information will be relevant to innovators developing new RMTs for management of different health challenges, including treatment of vulnerable and marginalized patients with COVID-19.

Meeting needs of vulnerable populations requires consideration of equity in the present and for future health delivery endeavors. Equity considerations are those that focus on ways to decrease or eliminate differences in health outcomes and opportunities across groups [13]. It is vital to incorporate an equity lens since COVID-19 is more likely to impact marginalized populations [14-16]. Intentional efforts to reduce inequity are important in health care design. Considerations in inequity have been conceptualized using the PROGRESS-Plus acronym, which is used to identify characteristics that stratify health opportunities and outcomes [17].

Thus far, published research has presented a selection of health and resource benefits and highlighted some challenges of using RMTs. However, no paper has comprehensively summarized the wide range of advantages and challenges of technologies used to deliver health care in the context of the COVID-19 pandemic. Further, there are no reviews that specifically address equity of access to health care in the context of COVID-19.

A comprehensive review and summary of RMTs, including their advantages and challenges, together with future directions for innovation or improvement, will be a valuable asset for stakeholders. This information can be used to understand the breadth of available RMT options, anticipate and prevent difficulties, and improve equitable access to health care. This is particularly relevant early in the course of a pandemic when time-sensitive information is required.

The aim of this rapid review was to identify which RMTs have been deployed to support COVID-19 health care provision early in the pandemic, and identify the barriers to and benefits of their implementation. The review will emphasize equity considerations.

## Methods

A rapid review was performed following the approach described by Cochrane Methods Group [18]. Rapid reviews offer a strategy to synthesize current knowledge in a timely manner “to meet time-sensitive decision-making needs” [1], which is valuable in contexts such as the COVID-19 global health crisis.

### Search Methods

Ovid MEDLINE and Embase were searched for relevant publications published up until July 6, 2020. The search was initially informed by a preliminary unstructured search of QxMD [19] and MEDLINE (via PubMed). The search strategy (Multimedia Appendix 1) was developed using the COVID-19 keywords from the Canadian Agency for Drugs and Technologies in Health search strings [12].

### Inclusion Criteria

Inclusion criteria for selection of publications were as follows: publications that discussed use of RMTs to deliver health care to patients with COVID-19; publications that referred to specific RMTs as opposed to general recommendations (this criterion was used to ensure barriers could be linked to a specific type of technology); and publications that were available in English, French, or Spanish.

### Exclusion Criteria

Publications were excluded if the record only described RMTs used in contact tracing or epidemiological surveillance. This criterion was used because the focus of this review was on technologies that provide service or treatment to patients with COVID-19, rather than surveillance of the virus.

### Search Strategy

An inclusive search strategy for different types of publications was used and included editorials, reviews, and letters to the editor. This inclusive strategy was adopted considering the novelty of COVID-19 (and hence dearth of published research-based studies). Further, the inclusive search strategy reflected the potential benefits of presenting a breadth of information at this early stage of the pandemic.

### Data Collection

The primary author (EH) reviewed the title and abstract of publications then extracted key information. Key information extracted included the year, journal, design, aim of the publication, nature of the RMT, whether the RMT was synchronous or asynchronous, information gathered via RMT, equity considerations, benefits of RMT use, and barriers to RMT use.

### Data Analysis

Publications’ results were synthesized using a “content analysis” approach [20]. A single reviewer (EH) developed a coding framework and mapped the RMTs used and variables monitored. Barriers and benefits of using RMT were coded inductively, and individual codes were grouped into overarching themes. A second reviewer (DOP) independently reviewed the developed coding framework against a random sample (7/48, 15%) of the publications. Further, the second reviewer coded this sample of publications against the finalized coding framework. All discrepancies (7% discrepancy rate) in the coding framework or coding were settled by consensus-based discussion.

To ensure explicit consideration of health equity, relevant considerations were mapped deductively to the PROGRESS-Plus acronym [17]. The PROGRESS-Plus acronym refers to consideration of the following: place of residence, race or ethnicity or culture or language, occupation, gender or sex, religion, education, socioeconomic status, social capital, and “plus”—referring to personal characteristics associated with discrimination, features of relationships, and time-dependent relationships [17].

## Results

### Results of Database Search

**Figure 1:**
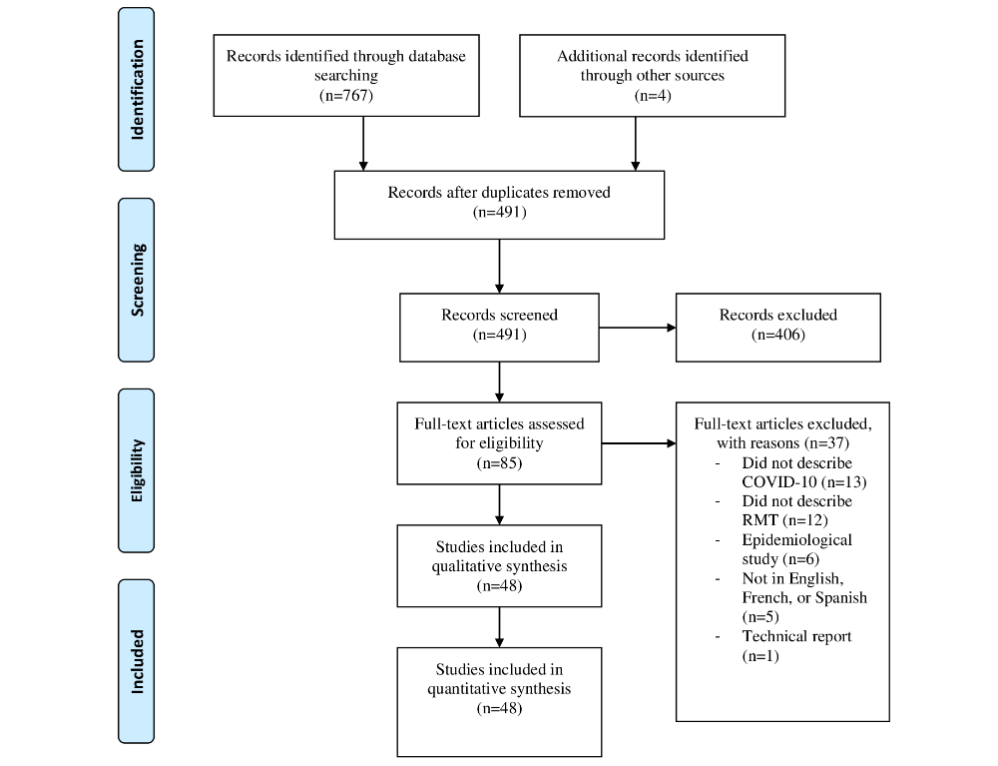
PRISMA 2009 flow diagram. PRISMA: Preferred Reporting Items for Systematic Reviews and Meta-Analyses; RMT: remote monitoring technology.

Of 767 records identified through MEDLINE and Embase searches, and 4 records identified through QxMD, 491 remained after duplicates were removed (Figure 1). After title and abstract screening, 406 records were excluded from the review. Upon full-text review, 37 of 85 records were excluded because they did not include health care for patients with COVID-19 (n=13), did not describe an RMT (n=12), were epidemiological studies and/or only described contact tracing (n=6), were not in English, Spanish, or French (n=5), or were technical reports (n=1). In total, 48 publications were included in the qualitative synthesis.

### Characteristics of Included Publications

The most common types of publications identified were descriptive studies or proposals describing the implementation and development of RMTs (n=18) and reviews of multiple RMTs (n=13). Letters to the editor (n=9), editorials (n=5), one retrospective cohort study, one protocol, and one case study were also included (Table 1).

**Table 1:** Publication types and remote monitoring technologies included in the analysis.

Publication type and ID		First author [reference]	Remote monitoring technologies
**Implementation and development**			
	2	Annis [21]	Mobile app
	4	Bae [22]	Chat or telephone consultation, digital blood pressure monitor, digital thermometer, digital pulse oximeter
	10	Faezipour [23]	Mobile app diagnostic test
	13	Ford [24]	Chat or telephone or video consultation, data transmission, digital pulse oximeter, digital thermometer, electronic health record, mobile app, patient-accessed electronic health record
	25	Krausz [25]	Chat or video consultation, electronic health record, mobile app, patient-accessed electronic health record, Voice over Internet Protocol technology, websites
	26	Lam [12]	Video consultation
	27	Lui [26]	Electronic health record, mobile apps
	28	Mann [27]	Electronic health record, video consultation
	30	Medina [28]	E-prescription, mobile app, telephone consultation
	32	Naik [29]	Closed-circuit television cameras, digital blood pressure monitor, digital electrocardiogram or heart rate monitor, digital end-tidal carbon dioxide monitor, digital pulse oximeter, digital respiratory rate monitor, mobile device video
	34	Petrocelli [30]	Home diagnostic test
	35	Saleem [31]	Automated SMS text messaging program
	36	Schinköthe [32]	Electronic health record, mobile app
	39	Song [33]	Video consultation
	40	Sossai [34]	Chat or video consultation, mobile app
	42	Timmers [35]	Mobile app
	44	Vaira [36]	Home diagnostic test, telephone consultation
**Implementation and development proposal**			
	38	Sharma [37]	Wearable devices or biometric clothing
**Retrospective cohort study**			
	47	Xu [38]	Chat or telephone consultation
**Case study**			
	20	Huang [39]	Chat consultation, mobile app
**Review**			
	1	Alwashimi [40]	Artificial intelligence–enabled request for assistance, chatbots, digital stethoscope, drone-delivered home diagnostic tests, glucometer, mobile app smart inhaler, video consultation, wearable devices or biometric clothing, website
	7	Crawford [41]	Video consultation
	8	Ding [10]	Digital electrocardiogram or heart rate monitor, digital blood pressure monitor, digital respiratory rate monitor, digital pulse oximeter, digital stethoscope, digital thermometer, mobile device recording, wearable devices or biometric clothing
	11	Fagherazzi [42]	Chat or telephone or video consultation, e-prescription
	18	Hong [43]	E-prescription, mobile phone app, telephone consultation, video consultation
	19	Horowitz [44]	Mobile app diagnostic test, mobile app
	23	Kannampallil [45]	Mobile app, wearable devices or biometric clothing
	24	Keshvardoost [46]	Electronic health record, telephone or video consultation
	29	Massaroni [47]	Laptop camera, mobile device camera, radar/Wi-Fi transmitter-receiver, smart mattress, tablet camera, wearable devices or biometric clothing
	31	Ming [48]	Mobile app
	37	Sharma [49]	Digital blood pressure monitor, digital electrocardiogram or heart rate monitor, digital glucometer, digital pulse oximeter, digital posture, digital respiratory rate monitor, digital temperature monitor, digital weight monitor, patient-accessed electronic health record, video consultation
	41	Thulesius [50]	Video consultation
	45	Watson [51]	Home diagnostic test, mobile app, video consultation
	48	Ye [52]	Telephone or video consultation
**Editorial**			
	6	Cohen [53]	Tablet app, telephone consultation
	9	Edelman [54]	Electronic health record, video consultation
	16	Grenngalgh [55]	Video consultation
	22	John [56]	Video consultation
**Protocol**			
	15	Greenhalgh [57]	Telephone or video consultation
**Letter to the editor**			
	3	Anonymous [58]	Telephone consultation
	5	Barsom [59]	Electronic health records, patient-accessed electronic health record, tablet, video consultation
	12	Fitz [60]	Chat consultation
	14	Giansanti [61]	Mobile app, video consultation
	17	Hau [62]	Telephone or video consultation
	21	Jamil [63]	Telephone or video consultation
	33	Nair [64]	Electronic health record mobile app, telephone or video consultation
	43	Trethewey [65]	Telephone or video consultation
	46	Wei [66]	Video consultation

### Description of RMTs

These publications identified 35 distinct types of RMTs (32 asynchronous and 3 synchronous; Table 2). Overall, 35 of the 48 studies identified synchronous technologies, including video (n=25), telephone (n=15), chat (n=8), and mobile or cellular device video (n=3) consultations. In total, 33 studies reported asynchronous technologies, including mobile or cellular apps for patients to manually enter their symptoms or education (n=17), home diagnostic tests (n=6), remote access to electronic health records by provider (n=8), digital pulse oximeters (n=5), biometric clothing (n=5), and remote access to electronic health records by patient (n=5). Data were collected through manual input by patients, caregivers, and health care practitioners (n=45; Multimedia Appendix 2), and automatically through the use of biosensor technology (n=22; Multimedia Appendix 3). The most commonly reported manually inputted outcome measures were “symptoms” (n=18), dyspnea or shortness of breath (n=7), pulse oximetry (n=7), mental health (n=7), temperature (n=6), and presence or absence of fever (n=6). The most frequently reported biosensor measures were temperature (n=5) and cardiac activity (as measured by an electrocardiogram; n=5).

**Table 2:** Summary of remote monitoring technologies grouped into synchronous or asynchronous.

Type of remote monitoring technology		Total publications, n
**Asynchronous**		
	Mobile or cellular app	17
	Electronic health record (eg, cloud based)	8
	Home diagnostic tests	6
	Digital (eg, Bluetooth) pulse oximeter	5
	Wearable device or biometric clothing	5
	Patient-accessed electronic health record	5
	Digital thermometer	4
	Digital blood pressure sensor	4
	Mobile or cellular app diagnostic test (acoustic signal, olfactory and gustatory test)	3
	Digital heart rate monitor, respiratory rate monitor, and electrocardiogram (eg, measure heart rate variability)	3
	Tablet (eg, iPads) may be used for video	3
	E-prescription	3
	Website to monitor symptoms	2
	Digital stethoscope	2
	Glucometer	2
	Data transmission technology	2
	Automated SMS text messaging program	1
	Chatbot, drone, artificial intelligence–enabled request for assistant, smart inhaler	1
	Closed-circuit television camera and digital end-tidal carbon dioxide monitor	1
	Digital weight and posture sensors	1
	Smart mattress	1
	Voice over Internet Protocol technology	1
**Synchronous**		
	Video consultation	25
	Telephone consultation	15
	Chat consultation	7
	Mobile or cellular device video consultation	2

### Benefits of RMTs

Almost all publications (46/48, 96%) provided some description of the reported benefits of RMTs. In total, 34 distinct codes were developed inductively from these documents (Multimedia Appendix 4). These were grouped into 10 themes (Table 3): “reduces infection risk” (n=33 articles), “reduces burden of care” (eg, limits hospital beds used; n=28), “supports vulnerable populations” (n=14), “reduces costs” (n=12), “improves patient experience” (n=11), “promotes knowledge development” (n=8), “facilitates navigation through health care system” (n=8), “improves health outcomes” (n=6), “supports public health initiatives” (n=5), and “technology-specific benefits” (n=4).

The most common theme, “reduces infection risk,” included 3 codes: “reduces risk of transmission generally” (n=18), “reduces exposure of health care practitioners” (n=11), and “reduces cross-contamination or clustering” (n=10). A total of 28 codes were mapped to the theme “reduces burden of care,” notably that RMTs “reduce the burden on hospitals” (eg, limit hospital beds used; n=17), “provide continuous accessible data or monitoring” (n=12), and “reduce burden of time on health care workers” (eg, using asynchronous technology or reducing burden overall; n=6). In addition, 5 codes were mapped to the theme “RMTs support vulnerable populations,” including “reduces need for transfer of vulnerable patients” (n=6) and “supports patient mental health” (eg, relieves stress, supports anxious patients; n=5). A total of 3 codes were mapped to the theme “reduces cost,” notably “reduces health care system or public health agency costs” (n=12). A total of 3 codes were mapped to the theme “improves patient experience,” including “improves patient initiative, engagement, autonomy, or self-management” (n=5). In addition, two codes were mapped to “facilitates navigation through the health care system,” notably “facilitated follow-up, continuity of care, or linkage to care” (n=5). The theme “improves health outcomes” contained only one code, “provides rapid identification of infection or clinical deterioration for timely treatment of COVID-19” (n=6). Finally, the theme “supports public health initiatives” contained 2 codes, including “delivers educational messages” (eg, fights disinformation or the “infodemic”; n=5). All other codes were noted by <5 publications.

**Table 3:** Perceived benefits of remote monitoring technologies for patients with COVID-19.

Themes	Total publications, n
Reduces infection risk	33
Reduces burden of care	28
Supports vulnerable populations	14
Reduces costs	12
Improves patient experience	11
Promotes knowledge development	8
Facilitates navigation through health care system	8
Improves health outcomes	6
Supports public health initiatives	5
Technology-specific benefits	4

### Barriers to Using RMTs

Many publications (38/48, 80%) reported barriers, challenges, and/or concerns regarding implementation of RMTs (Table 4). In all, 54 distinct codes were developed inductively (Multimedia Appendix 5) and mapped to 15 themes: “equity-related barriers” (n=16), “a lack of RMT implementation guidelines and research” (n=12), “resources required for technology development and implementation” (n=11), “challenging patient experiences of RMTs” (n=10), “confidentiality-related concerns” (n=10), “workforce training” (n=8), “quality of information” (n=8), “communication-related barriers” (n=7), “ethical concerns with RMTs” (n=7), “policy requirements” (n=7), “quality of care concerns” (n=4), “technology-specific barriers” (n=3), “technology integration–related barriers” (n=2), and “financial barriers” (n=2). A total of 8 codes were mapped to the theme “equity-related barriers,” including “lack of access to RMTs in low-resource settings” (eg, patients experiencing homelessness, neighborhoods without access to libraries, households without internet or devices, low-income communities unable to afford RMTs or share RMTs; n=8), “low network quality or internet connectivity or bandwidth,” which can impact quality of care (n=6), and “low patient health literacy” (n=5). A total of 5 codes were mapped to the theme “a lack of RMT implementation guidelines and research,” with 9 publications reporting a “paucity of high-quality data or guidelines to support effective and safe RMT, particularly in acute care.” A total of 7 codes were mapped to the theme “resources required for technology development and implementation,” including “inadequate control of patient flow with some RMTs” (eg, the fluctuating recruitment of patients should be matched with staffing; n=5). A total of 4 codes were mapped to the theme “challenging patient experiences of RMTs,” including “the complexity or intrusiveness of switching to online consultation or remote monitoring and disruption to patient or worker processes and routines” (n=5). A total of 3 codes were mapped to the theme “confidentiality-related barriers,” including “the need to address privacy concern when implementing RMTs” (n=9). A total of 3 codes were mapped to the theme “workforce training,” including “the need for additional workforce education and training in use of RMTs” (n=6). A total of 2 codes were mapped to the theme “quality of information,” including “issues regarding the quality of health information reported or collected” (eg, self-reporting; n=6). Finally, 3 codes were mapped to the theme “ethical concerns with RMTs,” of which the majority (n=5) of the publications reported general ethical concerns. All other codes were reported by <5 publications.

**Table 4:** Perceived barriers to using remote monitoring technologies for patients with COVID-19.

Themes	Total publications, n
Equity-related barriers	16
A lack of remote monitoring technology implementation guidelines and research	12
Resources required for technology development and implementation	11
Challenging patient experiences of remote monitoring technologies	11
Confidentiality-related barriers	10
Workforce training	8
Quality of information	8
Communication-related barriers	7
Ethical concerns with remote monitoring technologies	7
Policy requirements	7
Quality of care	4
Technology-specific barriers	3
Technology integration–related barriers	2
Financial barriers	2

### Equity Factors

Equity groups were mapped deductively to the PROGRESS-Plus acronym (Table 5). Several publications (n=23) noted how remote monitoring provided information on characteristics of populations that could stratify health opportunities and outcomes (Multimedia Appendix 6). The most frequently reported characteristic was the “Plus” code (n=17). This “Plus” code refers to specific patient populations (ie, patients who distrust the health care system, those with chronic conditions or comorbidities, immune-suppressed patients, pregnant women, acutely unwell patients, patients with cognitive impairment, nonadherent patients, seniors, and youth). The next most common PROGRESS-Plus code was “place of residence.” A total of 11 publications commented on patient place of residence as impacting the implementation of RMTs, including rural and remote residences, nursing homes, or homelessness. A total of 7 publications reported socioeconomic factors, such as a lack of community funding in low- and middle-income countries, lack of access to technology, or low-income patients. A total of 3 publications reported on race or ethnicity or culture or language, occupation, and gender or sex, respectively. Occupations considered were frontline workers and veterans. Finally, in terms of PROGRESS-Plus equity considerations, 2 publications commented on education, specifically the health literacy level of patients, which could impact their ability to understand infection control information or assess the quality of unregulated health information. Only one publication commented on social capital and none on religion.

**Table 5:** PROGRESS-Plus themes.

Theme	Total publications, n	Demonstrative quote
Place of residence	11	“It is also important to consider that some countries may not have the technological infrastructure to support [digital health]. Furthermore, there will be a significant proportion of the population who will not have access to technology or internet connectivity” [40]
Race or ethnicity or culture or language	3	“Mounting evidence suggests that the COVID-19 pandemic has far greater associated morbidity and mortality in racialized groups that struggle with poverty and poor access to health care; the pandemic has also been suggested to compound pre-existing inequities. Similarly, there has been a lack of attention to health equity in the development of digital health solutions” [41]
Occupation	3	“Individuals exposed to the public (such as transit workers and police/force/emergency medical services (EMS) workers) and cultural settings with risk for infection (eg, multifamily settings with multiple house members working with high risk for COVID-19 exposure settings), in addition to those in meat packing plants or the front-line grocery store workers, are especially in the high-risk exposure category. This additionally emphasizes the need for developing testing models of the breathing app from the breathing sound database.” [23]
Gender or sex	3	“In Dover and Belon’s model, which informs the foundation of the [Digital Health Equity Framework], the process of social stratification within economic and cultural social contexts refers to the hierarchical allocation and unequal distribution of power, prestige, and resources; this stratification assigns individuals to a social location, which is defined by intersectional factors such as race, age, income, geography, rurality, gender, ability, and occupation as well as other social factors” [41]
Religion	0	—^a^
Education	2	“Our findings suggest that even in high-income countries, such as the [United Arab Emirates] with modern digital access and high general literacy rates, health literacy may pose an obstacle for the adoption of telemedicine. It is, therefore, critical for countries worldwide to improve health literacy to optimize patient access and engagement in this expanding world of digital medical care delivery.” [64]
Socioeconomic status	7	“Lastly, some developing countries face major obstacles to the effective delivery of digital health solutions in rural and remote locations, such as incomplete or insufficient basic digital infrastructures (eg, computers, internet networks, and electricity), lack of sustainable funding to develop, operate, and maintain digital platforms, and high telecommunication costs” [42]
Social capital	1	See “Gender or sex” above [41]
Plus	17	“The network has focused on groups particularly vulnerable to severe symptoms of COVID-19, including the elderly, pregnant women, children, and patients with chronic health problems.” [43]

^a^Not available.

## Discussion

### Principal Findings

A rapid review was conducted to present the different types of RMTs deployed in response to the COVID-19 pandemic, the perceived benefits of these technologies, and the perceived barriers or challenges to their use in clinical work. There was special emphasis placed on equity considerations.

Results indicate that many different RMTs are used when providing health care to patients with COVID-19. Most RMTs mirrored the real-time interpersonal communication processes involved in prepandemic care, except that the care was being delivered with physical distance—for instance, the use of videoconferencing (25/48, 52%) and teleconferencing (15/48, 31%) rather than in-person appointments, and accessing medical records remotely (8/48, 17%) rather in the office. Video was likely favored because of the substantial advantage of being able to use nonverbal cues and assessment. These innovations were pertinent to monitoring, triage, and diagnosis (eg, symptom tracking using mobile apps).

### Benefits of RMTs and Implications

The most common reported benefit of RMTs was that it reduced the risk of transmission of COVID-19 (n=18), including to health care practitioners (n=10). This is unsurprising, considering that concern about managing infection rates is one of the forces driving rapid implementation of RMTs across many specialties [67]. Further, with increasing pressure on the health care system, RMTs could aid in reducing the burden on hospitals (n=17), health care system costs (n=10), and the burden of time of health care practitioners (n=16).

Use of these RMTs began or substantially increased in response to COVID-19. If perceived advantages are borne out, this may lead to wider adoption beyond the pandemic.

There may be several implications when considering the breadth of RMTs already in use. First, both health care providers and patients may become accustomed to the remote modality and its benefits, including increased convenience, meaning that RMT use could continue beyond the duration of the pandemic. Health care during the pandemic has increased patient involvement in their own care (eg, symptom monitoring). This may lead to a permanent shift in health care culture, in which patients’ active participation is incorporated into the health provision of other conditions. The development of health-related software for signs and symptom monitoring and triage could also continue and become refined and expand the tools through which quality health care is delivered. When considering whether or which changes might endure, it might be helpful to distinguish between advantages of RMTs that are only relevant to COVID-19 infection risk and those where the benefits might persist beyond COVID-19 (eg, widespread use of health-related apps to better monitor symptoms for other conditions). It is possible and likely that the processes surrounding their use will be refined to address and reduce concerns and barriers.

### Barriers to Use of RMTs

The most commonly reported barrier themes were a lack of guidance (n=12) and increased resources needed (n=11) for implementation, development, and use of RMTs to treat COVID-19. These barriers are likely due to the novel nature of COVID-19 and thus could become less relevant over time. Another main concern cited by several publications was that rigorous privacy and security settings would be necessary to protect patient information (n=9). However, despite emphasizing the importance of privacy and security, only 5 publications of 18 describing implementation of a specific RMT reported on security and privacy features or policies of the software used [24-26,32,35]. Additionally, two publications noted that use of RMTs could break down the humanitarian core of care as well as patient-provider communication [43,50]. One way to promote effective patient communication is to design user-friendly technology with two-way communication [68]. Although patient involvement in technology development can be used to effectively tailor the technology to the specific needs of the patient [69], no publications reported this in practice. This is unfortunate considering the impact it can have on the success of an RMT [70]. Future technologies should involve rigorous user evaluation based on feedback from patients. The extent of patient involvement in RMT implementation should be thoroughly described to support use of the technology. Lastly, it will take time and resources to bring RMTs to scale; information regarding clinical utility and cost will help ascertain which should be prioritized for investment of resources to aid in this development.

### Equity Factors

Equity factors also proved important to consider when implementing RMTs. Health interventions should be tailored according to population needs or they risk increasing health inequities [17]. The most common overall barrier theme was concerns regarding equitable use of RMTs (n=15). This is concerning considering marginalized groups are already disproportionately impacted by COVID-19. There is a higher incidence of infection, as well as poorer outcomes, among racialized communities and ethnic minorities [71-73]. More than half of the included publications (n=28) noted challenges that might be faced by minorities or emphasized the importance of training health practitioners in equitable digital health implementation and supporting low-income communities. Alternatively, possible benefits were reported in some papers (n=7), including the ability to support vulnerable populations—for example, medically vulnerable individuals, or those living in rural or remote communities. Overall, there appeared to be conflicting perspectives on whether RMTs would decrease or increase health inequities. There is evidence that intervention-generated inequities—those caused by use of interventions that provide limited benefit to vulnerable populations—can decrease health outcomes among marginalized groups [13]. It is important to track characteristics that may stratify health outcomes in order to assess which RMTs will benefit vulnerable populations. Overall, 23 of 48 total papers (48%) and 7 of 18 (39%) implementation and development papers reported on characteristics of populations that stratify health outcomes. It is critical that an increasing number of publications start to analyze data based on characteristics that influence equitable outcomes to better support vulnerable groups and reduce health inequities.

### Limitations

This work has several time-related limitations due to the rapid nature of this review. Only one screener (EH) reviewed the titles, abstracts, and full texts of the records and coded during the selection process. Further, given the delay between the use of an intervention and publication of its evaluation, the technologies presented here only reflect what was used during a specific window of time and as new technologies are rapidly developed and implemented, there will be a need to reassess new and emerging benefits of and barriers to patient care. Further periodic reviews should be conducted to assess how the use of RMTs evolves. In addition, the quality of the analysis was limited. Furthermore, the frequency of reporting does not necessarily equal the magnitude of importance or the impact on outcomes for patients. Lastly, certain papers reported on multiple technologies but did not provide benefits and barriers specific to each RMT individually, which limits the generalizability of our findings. As such, most benefits and barriers reported in this paper were categorized generally as applicable to both synchronous and asynchronous RMTs. Undoubtedly, many of these benefits and barriers will be more or less relevant depending on the technology (eg, reduced isolation is more applicable for treatment via video consultation than for use of a digital pulse oximeter). Future reviews should focus on separating benefits and barriers according to technology type if possible.

### Conclusion

This rapid review summarizes RMTs that were used in the early stages of the COVID-19 pandemic and provides insights on the benefits these technologies could provide for future use. It also highlights perceived barriers to implementation of RMTs that should be addressed in ongoing development projects. Guidelines and policies developed for implementing RMTs ought to be mindful of the identified barriers. RMTs have an important role to play in supporting patients and communities through these unprecedented times. One key recommendation is to establish best practices in the development of RMTs so they are both equitable and effective going forward.

## Appendix

Multimedia Appendix 1 COVID-19 remote monitoring search strategy: July 6, 2020.

Multimedia Appendix 2 Physiological parameters and symptoms that are manually inputted into remote monitoring technologies.

Multimedia Appendix 3 IDs of records that reported automatically recorded biosensor outcome measures.

Multimedia Appendix 4 IDs of records that reported benefits of remote monitoring technologies.

Multimedia Appendix 5 IDs of records that reported barriers to use of remote monitoring technologies.

Multimedia Appendix 6 IDs of records that identified characteristics that stratify health opportunities or outcomes mapped to the PROGRESS-Plus framework.

## Abbreviations

RMT: remote monitoring technology
