# Supplementary material for: Barriers to Use of Remote Monitoring Technologies Used to Support Patients With COVID-19: Rapid Review
Source: JMIR Mhealth Uhealth. 2021 Apr 20;9(4):e24743. doi: 10.2196/24743 (PMC8059785; doi:10.2196/24743)
Supplement: Multimedia Appendix 1 [file mhealth_v9i4e24743_app1.docx]

**Multimedia Appendix 1**

COVID-19 remote monitoring technology search strategy: Monday, July 6^th^, 2020

| Database and Coverage | Search Date | Results |
| --- | --- | --- |
| Ovid MEDLINE(R) ALL <1946 to July 02, 2020> | Monday, July 6^th^, 2020 | 348 |
| Embase Classic+Embase <1947 to 2020 July 02> | Monday, July 6^th^, 2020 | 419 |
| Total without duplicates | Monday, July 6^th^, 2020 | 486 |

# Medline

1 (coronavirus/ or betacoronavirus/ or coronavirus infections/) and (disease outbreaks/ or epidemics/ or pandemics/) (9842)

2 (nCoV* or 2019nCoV or 19nCoV or COVID19* or COVID or SARS-COV-2 or SARSCOV-2 or SARSCOV2 or Severe Acute Respiratory Syndrome Coronavirus 2 or Severe Acute Respiratory Syndrome Corona Virus 2).ti,ab,kf,nm,ot,ox,rx,px. (29079)

3 ((new or novel or "19" or "2019" or Wuhan or Hubei or China or Chinese) adj3 (coronavirus* or corona virus* or betacoronavirus* or CoV or HCoV)).ti,ab,kf,ot. (10306)

4 ((coronavirus* or corona virus* or betacoronavirus*) adj3 (pandemic* or epidemic* or outbreak* or crisis)).ti,ab,kf,ot. (2243)

5 ((Wuhan or Hubei) adj5 pneumonia).ti,ab,kf,ot. (184)

6 Hemorrhagic Fever, Ebola/ (5416)

7 ebola.ti,ab,kf,nm,ot,ox,rx,px. (8619)

8 1 or 2 or 3 or 4 or 5 or 6 or 7 (40395)

9 (eConsult* or e-consult* or ecounsel* or e-counsel* or eHealth* or e-Health* or einterv* or e-interv* or etherap* or e-therap* or mHealth* or m-Health* or mobile health*).ti,ab,kf. (14886)

10 (App or apps or facetime* or helpline* or skype* or zoom or webbased tool or web-based tool*).ti,ab,kf. (33114)

11 Remote Consultation/ (4749)

12 ((remote* or distan*) adj3 (consult* or eval* or assess* or monitor* or follow*)).tw,kf. (12041)

13 9 or 10 or 11 or 12 (60547)

14 8 and 13 (348)

# Embase

1 (coronavirinae/ or betacoronavirus/ or Coronavirus infection/) and (epidemics/ or pandemic/) (3005)

2 (nCoV* or 2019nCoV or 19nCoV or COVID19* or COVID or SARS-COV-2 or SARSCOV-2 or SARSCOV2 or Severe Acute Respiratory Syndrome Coronavirus 2 or Severe Acute Respiratory Syndrome Corona Virus 2).ti,ab,kw,ot,ox. (26147)

3 ((new or novel or "19" or "2019" or Wuhan or Hubei or China or Chinese) adj3 (coronavirus* or corona virus* or betacoronavirus* or CoV or HCoV)).ti,ab,kw,ot. (9503)

4 ((coronavirus* or corona virus* or betacoronavirus*) adj3 (pandemic* or epidemic* or outbreak* or crisis)).ti,ab,kw,ot. (2025)

5 ((Wuhan or Hubei) adj5 pneumonia).ti,ab,kw,ot. (188)

6 Ebola hemorrhagic fever/ (5843)

7 ebola.ti,ab,kw,ot,ox. (11632)

8 1 or 2 or 3 or 4 or 5 or 6 or 7 (39769)

9 (eConsult* or e-consult* or ecounsel* or e-counsel* or eHealth* or e-Health* or einterv* or e-interv* or etherap* or e-therap* or mHealth* or m-Health* or mobile health*).ti,ab,kw. (16327)

10 (App or apps or facetime* or helpline* or skype* or zoom or webbased tool or web-based tool*).ti,ab,kw. (46517)

11 teleconsultation/ (9556)

12 ((remote* or distan*) adj3 (consult* or eval* or assess* or monitor* or follow*)).tw,kw. (17634)

13 9 or 10 or 11 or 12 (85432)

14 8 and 13 (419)
