# Supplementary material for: Barriers to Use of Remote Monitoring Technologies Used to Support Patients With COVID-19: Rapid Review
Source: JMIR Mhealth Uhealth. 2021 Apr 20;9(4):e24743. doi: 10.2196/24743 (PMC8059785; doi:10.2196/24743)
Supplement: Multimedia Appendix 2 [file mhealth_v9i4e24743_app2.docx]

**Multimedia Appendix 2.** Physiological parameters and symptoms that are manually inputted into RMTs

| **Manually inputted outcomes (total publications)** | **Record Ids** |
| --- | --- |
| Relevant background information | |
| Presence of chronic condition or comorbidities  (e.g., COPD^a^, asthma, diabetes, heart failure) (5) | 15, 26, 30, 42, 44 |
| Age (4) | 1,39, 42, 44 |
| Patient history (onset of history) (4) | 15, 30, 36, 44 |
| Demographics (3) | 14, 27, 30 |
| Travel history (3) | 1, 15, 26 |
| Contact history (3) | 15, 26, 27 |
| Gender (2) | 42, 44 |
| COVID-19 status or diagnosis (2) | 13, 31 |
| Contact with health care practitioner in the last 7 days (2) | 15, 42 |
| Postal code (e.g., first 4 digits) (1) | 42 |
| Employment status (1) | 44 |
| Symptoms and vital signs | |
| ‘Symptoms’ (e.g., COVID-19) (19) | 2, 3, 12, 13, 15, 16, 17, 20, 24, 26, 27, 28, 30, 31, 40, 42, 44, 46, 47 |
| Dyspnea, or shortness of breath (8) | 2, 6, 15, 20, 28, 30, 35, 42 |
| Blood oxygen levels, saturation, or pulse oximetry (8) | 2, 6, 15, 19, 20, 30, 39, 45 |
| Mental health symptoms (e.g., stress, psychiatric symptoms) (8) | 14, 15, 17, 19, 20, 23, 30, 47 |
| Temperature (7) | 1, 6, 15, 20, 30, 39, 45 |
| Fever (body temperature >38^o^C) (6) | 6, 15, 28, 35, 42, 47 |
| Diarrhea (4) | 15, 20, 30, 42 |
| Heart Rate (4) | 19, 15, 23, 48 |
| Respiratory rate (4) | 19, 15, 39, 48 |
| Vital signs (3) | 19, 23, 36 |
| Flu-like symptoms (3) | 15, 28, 35 |
| Physical activity (e.g., ability to walk, ability to exercise) (3) | 15, 23, 26 |
| Blood pressure (3) | 6, 15, 39 |
| Weakness or lack of strength (2) | 20, 30 |
| Cough (2) | 20, 30 |
| Muscle soreness | 15, 20 |
| Pneumonia (2) | 15, 28 |
| Appetite (2) | 15, 30 |
| Pulse rate (2) | 15, 39 |
| Miscellaneous symptoms *(sole studies)* (vomiting, chest tightness, glucose levels, sinusitis, bronchitis, pharyngitis, cyanosis, sneezing, runny nose, level of consciousness, heart rate variability, measures of olfaction, olfactory and gustatory test, satisfaction) | 19, 20, 28, 30, 34, 39, 42 |

^a^ Chronic obstructive pulmonary disease
