# Supplementary material for: Barriers to Use of Remote Monitoring Technologies Used to Support Patients With COVID-19: Rapid Review
Source: JMIR Mhealth Uhealth. 2021 Apr 20;9(4):e24743. doi: 10.2196/24743 (PMC8059785; doi:10.2196/24743)
Supplement: Multimedia Appendix 3 [file mhealth_v9i4e24743_app3.docx]

**Multimedia Appendix 3.** Ids of records that reported automated recorded biosensor outcome measures

| **Outcome measure (Total publications)** | **Record Ids** |
| --- | --- |
| Temperature (5) | 1, 8, 13, 37, 38 |
| Electrocardiogram signals (5) | 1, 8, 19, 32, 38 |
| Blood oxygen level (4) | 8, 13, 32, 38 |
| Respiratory rate (4) | 8, 29, 32, 38 |
| Heart rate (4) | 8, 32, 37, 38 |
| Blood pressure (4) | 8, 32, 37, 38 |
| Galvanic skin response (2) | 8, 37 |
| Vital signs (2) | 1, 38 |
| Blood glucose levels (2) | 1, 38 |
| Lung acoustic signals (2) | 8, 10 |
| Miscellaneous symptoms *(outcomes reported in only one study):* Digitally monitored symptoms, Visualization of patient (and mechanical ventilation), Bioimpedance, Auscultation, Respiration effort, Pupil diameter, Weight, Posture, EtCO2^a^, Heart rate variability | 1, 8, 32, 37, 38  38 |

^a^ End-tidal carbon dioxide
