# Supplementary material for: Barriers to Use of Remote Monitoring Technologies Used to Support Patients With COVID-19: Rapid Review
Source: JMIR Mhealth Uhealth. 2021 Apr 20;9(4):e24743. doi: 10.2196/24743 (PMC8059785; doi:10.2196/24743)
Supplement: Multimedia Appendix 4 [file mhealth_v9i4e24743_app4.docx]

**Multimedia Appendix 4**: Ids of records that reported benefits of RMTs

| **Themes and code (total publications)** | **Record Ids** |
| --- | --- |
| **Reduces infection risk (33)** | 1, 2, 4, 7, 8, 9, 10, 11, 13, 14, 15, 16, 17, 20, 21, 23, 24, 25, 26, 27, 29, 32, 33, 34, 36, 37, 39, 40, 41, 43, 45, 46, 47 |
| Reduces risk of transmission (generally) (18) | 2, 7, 8, 11, 13, 14, 15, 16, 23, 24, 26, 34, 39, 40, 41, 43, 46, 47 |
| Reduces exposure of healthcare practitioners (11) | 1, 4, 13, 24, 25, 27, 29, 32, 33, 36, 37 |
| Reduces cross-contamination, or clustering (10) | 9, 10, 13, 17, 20, 21, 24, 25, 29, 45 |
| **Reduces burden of care (28)** | 5, 6, 7, 8, 11, 12, 13, 16, 18, 20, 21, 22, 24, 25, 27, 28, 29, 30, 31, 32, 36, 37, 39, 41, 42, 43, 45, 48 |
| Reduces burden on hospitals (e.g., limits hospital beds used) (17) | 5, 6, 7, 8, 11, 12, 18, 20, 22, 28, 30, 31, 32, 39, 42, 43, 45 |
| Provides continuous accessible data or monitoring (12) | 8, 11, 13, 18, 20, 25, 27, 29, 31, 32, 36, 42, 45 |
| Reduce burden of time on healthcare workers (e.g. using asynchronous technology, or reducing burden overall) (6) | 8, 13, 20, 25, 27, 32 |
| Reduces time burden on patients (4) | 21, 24, 25, 28 |
| Data collection is fluid with electronic health record (e.g., reduces data entry workload and increases capacity to manage dynamic information) (4) | 9, 24, 37, 45 |
| Technology supports clinical decision-making (e.g., using branching logic, decision aids) (3) | 9, 41, 48 |
| Allows clinicians to work offsite or when they are self-isolating (2) | 16, 32 |
| Reduces caregiver burden (1) | 30 |
| **Supports vulnerable populations (14)** | 7, 9, 15, 16, 18, 20, 23, 24, 25, 28, 30, 31, 33, 35 |
| Reduces need for mobility of vulnerable patients (6) | 16, 20, 24, 28, 31, 33 |
| Supports patient mental health (e.g., relieves stress, supports anxious patients) (5) | 7, 16, 18, 23, 30 |
| Supports individuals in rural and remote settings (3) | 23, 24, 25 |
| Promotes social interaction, feelings of connectedness, or reduces feelings of loneliness (3) | 9, 15, 35 |
| Flags, or identifies and supports vulnerable populations (2) | 9, 15 |
| **Reduces costs (12)** | 9, 12, 13, 18, 22, 24, 29, 30, 31, 32, 35, 45 |
| Reduces healthcare system, or public health agency costs (12) | 9, 12, 13, 18, 22, 24, 29, 30, 31, 32, 35, 45 |
| Reduced or no cost for patients (2) | 12, 45 |
| Reduces personal protective equipment use (1) | 9 |
| **Improves patient experience (11)** | 2, 8, 15, 18, 20, 25, 29, 30, 35, 41, 42 |
| Improves patient initiative, engagement, autonomy, or self-management (5) | 20, 25, 30, 35, 41 |
| Is convenient, user friendly, or unobtrusive (5) | 8, 15, 18, 29, 42 |
| Is associated with high patient satisfaction (3) | 2, 30, 42 |
| **Promotes knowledge development (8)** | 18, 23, 25, 29, 36, 42 44, 45 |
| Facilitates the implementation of program evaluation (4) | 18, 25, 36, 45 |
| Can inform COVID-19 treatment guidelines (4) | 23, 29, 42, 44 |
| Allows the standardization of questions (2) | 25, 45 |
| **Facilitates navigation through healthcare system (8)** | 2, 9, 14, 15, 17, 20, 25, 30 |
| Facilitates follow-up, continuity of care, or linkage to care (5) | 2, 14, 17, 20, 25, 30 |
| Facilitates referral (3) | 9, 15, 25 |
| **Improves health outcomes (6)** | 8, 15, 19, 31, 32, 42 |
| Provides rapid identification of infection, or clinical deterioration for timely treatment of COVID-19 (6) | 8, 15, 19, 31, 32, 42 |
| **Supports public health initiatives (5)** | 9 15, 35, 42, 45 |
| Delivers educational messages (fights disinformation, or ‘infodemic’) (5) | 9 15, 35, 42, 45 |
| Reduces burden on other public health agencies (1) | 35 |
| **Technology-specific benefits (4)** | 8, 13, 15, 45 |
| Video provides visual information and cues (3) | 13, 15, 45 |
| Blood pressure monitoring-specific advantages (1) | 8 |
| Electrocardiogram monitoring-specific advantages (1) | 8 |
| Telephone is familiar and dependable (1) | 15 |
