# Supplementary material for: Barriers to Use of Remote Monitoring Technologies Used to Support Patients With COVID-19: Rapid Review
Source: JMIR Mhealth Uhealth. 2021 Apr 20;9(4):e24743. doi: 10.2196/24743 (PMC8059785; doi:10.2196/24743)
Supplement: Multimedia Appendix 5 [file mhealth_v9i4e24743_app5.docx]

**Multimedia Appendix 5:** Ids of records that reported barriers to use of RMTs

| **Theme and code (total publications)** | **Record Ids** |
| --- | --- |
| **Equity-related barriers (16)** | 1, 5, 7, 9, 11, 13, 15, 16, 21, 24, 25, 26, 33, 42, 44, 48 |
| Lack of access to RMTs in low resource settings (homeless patients, neighbourhoods without access to libraries, households without internet or devices, low-income communities unable to afford RMTs or share RMTs) (8) | 1, 7, 11, 16, 21, 24, 25, 33 |
| Low network quality or internet connectivity or bandwidth which can impact quality of care (6) | 1, 9, 13, 15, 24, 48 |
| Low patient health literacy (5) | 5, 7, 11, 21, 33 |
| The higher level of patient compliance or cooperation often required by remote monitoring (2) | 42, 44 |
| A lack of personal or private space to engage in telehealth at home (2) | 1, 7 |
| Possible reduced uptake (technology less likely to be used or understood) by vulnerable populations, minorities, the elderly, or those living in rural or remote communities (2) | 11, 26 |
| Negative beliefs about RMTs (e.g., it is harmful, not valuable to care, or unfavorable for cultural or religious or moral reasons) (2) | 7, 11 |
| Lack of access to RMT among homeless patients (2) | 7, 21 |
| **A lack of RMT implementation guidelines and research (12)** | 1, 10, 15, 16, 22, 24, 25, 26, 28, 30, 43, 47 |
| The paucity of high-quality data or guidelines to support effective and safe RMT, particularly in acute care (9) | 10, 15, 16, 22, 24, 26, 30, 43, 47 |
| Difficulty identifying which patients may not be suitable candidates for remote monitoring (e.g., physical examination needed) (2) | 16, 43 |
| Difficulties related to rapid implementation of RMT (particularly in the context of a crisis) (2) | 16, 25 |
| Concern that it will be difficult to reverse regulations or expectations that are specific to the pandemic (1) | 28 |
| Barriers in program evaluation of RMTs (heterogenous implementation) (1) | 1 |
| **Resources required for technology development and implementation (11)** | 1, 2, 5, 7, 13, 16, 25, 26, 27, 29, 30 |
| Inadequate control of patient flow with some RMTs (e.g., fluctuating recruitment of patients needs to be matched with staffing) (5) | 2, 16, 25, 26, 30 |
| The need for capacity building, as many existing RMTs are not built to scale, are unable to accommodate surges in demand, are resource intensive and/or are costly (4) | 1, 5, 13, 25 |
| The need for additional time and resources for data processing and storage (2) | 7, 29 |
| Challenges (time, resources, access to relevant data) integrating RMTs into existing electronic health records systems (2) | 25, 27 |
| The absence of built-in analytic engines in some RMTs which prevents access to relevant information in a timely manner (i.e. live visualization or reporting data) (1) | 25 |
| The need for time and resources to develop an application program interface (1) | 25 |
| The need to develop RMTs within the confines of device power consumption (1) | 8 |
| **Challenging patient experiences of RMTs (11)** | 5, 7, 8, 11, 13, 16, 20, 23, 26, 35, 40 |
| The complexity or intrusiveness of switching to online consultation or remote monitoring and disruption to patient or worker processes and routines (5) | 5, 11, 16, 35, 40 |
| Limitations related to ease of use for patients (particularly among symptomatic patients) (3) | 13, 23, 26 |
| Discomfort of RM wearable devices (2) | 7, 8 |
| Poor mental health outcomes associated with home quarantine (loneliness, anxiety, depression, fear, uncomfortableness) (1) | 20 |
| **Confidentiality-related barriers (10)** | 1, 11, 15, 16, 25, 31, 32, 38, 41, 48 |
| The need to address privacy concerns when implementing RMTs (9) | 1, 11, 16, 25, 31, 32, 38, 41, 48 |
| The need to address cybersecurity risks when implementing RMTs (2) | 31, 32 |
| The need to ascertain patient identity (1) | 15 |
| **Workforce training (8)** | 1, 5, 7, 11, 13, 16, 27, 43 |
| The need for additional workforce education and training in use of RMTs (6) | 1, 5, 7, 13, 16, 43 |
| A lack of workforce training in digital health equity or lack of cultural competence to understand how patients and communities experience or interact with technology (specific patient counselling should be implemented) (3) | 7, 11, 27 |
| The need for worker authorization and access (1) | 13 |
| **Quality of information (8)** | 8, 16, 18, 26, 27, 30, 41, 42 |
| Issues regarding the quality of health information reported or collected (e.g. self-reporting) (7) | 8, 16, 18, 26, 27, 30, 42 |
| Increased predictive or diagnostic uncertainty with at-home tests (1) | 41 |
| **Communication-related barriers (7)** | 15, 16, 18, 26, 30, 35, 41 |
| A lack of two-way communication in RMTs may leave patients feeling isolated, result in poor health outcomes or differing expectations for care (3) | 30, 35, 41 |
| Barriers associated with troubleshooting RMT malfunctions (2) | 15, 16 |
| Breakdown in the relationship between healthcare professionals and their patients which jeopardizes the humanitarian aspect of primary care (2) | 18, 41 |
| Breakdown in communication between healthcare professionals (interdisciplinary communication) (1) | 18 |
| Administrative and organizational challenges in scheduling virtual visits which are often on an impromptu basis (1) | 26 |
| **Ethical concerns with RMTs (7)** | 11, 14, 16, 25, 31, 41, 48 |
| Ethical concerns with RMTs (e.g., may erode personal freedoms) (5) | 11, 14, 31, 41, 48 |
| Possible trade-offs between thorough examination and reduced risk of infection (1) | 16 |
| A lack of patient access to their own data with many technologies (1) | 25 |
| **Policy requirements (7)** | 4, 5, 15, 20, 28, 31, 41 |
| Country-specific policies that limit RMTs (4) | 4, 15, 28, 41 |
| Licensing requirements (1) | 5 |
| The challenges or risk of spread of disinformation with unregulated RMT (1) | 31 |
| The need for patients to strictly adhere to the quarantine to avoid familial clustering (1) | 20 |
| **Quality of care (4)** | 7, 12, 16, 25 |
| Limitations in quality of care delivery (especially regarding screening tools) (2) | 16, 25 |
| Reduced linkage to care and increased loss to follow-up (1) | 12 |
| Use of digital health technologies for health avoidance (1) | 7 |
| **Technology-specific barriers (3)** | **7, 8, 15** |
| Digital respiratory rate monitoring-specific barriers (2) | 8, 15 |
| Electrocardiogram-specific barriers (1) | 7 |
| Digital pulse oximetry-specific challenges (1) | 8 |
| **Technology integration related barriers (2)** | 7, 25 |
| The multitude of RMT are associated with disparate health care systems and are not integrated into community and health infrastructure (2) | 7, 25 |
| **Financial barriers (2)** | **5, 7** |
| RMTs may not be covered by insurance provider (2) | 5, 7 |
