# Supplementary material for: Barriers to Use of Remote Monitoring Technologies Used to Support Patients With COVID-19: Rapid Review
Source: JMIR Mhealth Uhealth. 2021 Apr 20;9(4):e24743. doi: 10.2196/24743 (PMC8059785; doi:10.2196/24743)
Supplement: Multimedia Appendix 6 [file mhealth_v9i4e24743_app6.docx]

Multimedia Appendix 6: Ids of records that identified characteristics that stratify health opportunities or outcomes mapped to the PROGRESS plus framework

| Category (Total publications) | Record Ids |
| --- | --- |
|  |  |
| Place of residence (11) | 1, 7, 8, 9, 11, 13, 21, 23, 24, 33, 48 |
| Race or ethnicity or culture or language (3) | 7, 13, 33 |
| Occupation (3) | 7, 10, 35 |
| Gender or sex (3) | 7, 30, 47 |
| Religion (0) | - |
| Education (2) | 21, 33 |
| Socioeconomic status (7) | 1, 7, 11, 13, 33, 48 |
| Social capital (1) | 7 |
| Plus (17) | 7, 8, 9, 10, 14, 16, 17, 18, 21, 22, 24, 30, 33, 36, 43, 44, 47 |
